# Perturbations in the Photosynthetic Pigment Status Result in Photooxidation-Induced Crosstalk between Carotenoid and Porphyrin Biosynthetic Pathways

**DOI:** 10.3389/fpls.2017.01992

**Published:** 2017-11-20

**Authors:** Joon-Heum Park, Lien H. Tran, Sunyo Jung

**Affiliations:** BK21 Plus KNU Creative BioResearch Group, School of Life Sciences and Biotechnology, Kyungpook National University, Daegu, South Korea

**Keywords:** carotenoid, norflurazon, oxyfluorfen, photooxidation, porphyrin, rice

## Abstract

Possible crosstalk between the carotenoid and porphyrin biosynthetic pathways under photooxidative conditions was investigated by using their biosynthetic inhibitors, norflurazon (NF) and oxyfluorfen (OF). High levels of protoporphyrin IX (Proto IX) accumulated in rice plants treated with OF, whereas Proto IX decreased in plants treated with NF. Both NF and OF treatments resulted in greater decreases in MgProto IX, MgProto IX methyl ester, and protochlorophyllide. Activities and transcript levels of most porphyrin biosynthetic enzymes, particularly in the Mg-porphyrin branch, were greatly down-regulated in NF and OF plants. In contrast, the transcript levels of *GSA, PPO1*, and *CHLD* as well as *FC2* and *HO2* were up-regulated in NF-treated plants, while only moderate increases in *FC2* and *HO2* were observed in the early stage of OF treatment. Phytoene, antheraxanthin, and zeaxanthin showed high accumulation in NF-treated plants, whereas other carotenoid intermediates greatly decreased. Transcript levels of carotenoid biosynthetic genes, *PSY1* and *PDS*, decreased in response to NF and OF, whereas plants in the later stage of NF treatment exhibited up-regulation of *BCH* and *VDE* as well as recovery of *PDS*. However, perturbed porphyrin biosynthesis by OF did not noticeably influence levels of carotenoid metabolites, regardless of the strong down-regulation of carotenoid biosynthetic genes. Both NF and OF plants appeared to provide enhanced protection against photooxidative damage, not only by scavenging of Mg*-*porphyrins, but also by up-regulating *FC2, HO2*, and Fe-chelatase, particularly with increased levels of zeaxanthin via up-regulation of *BCH* and *VDE* in NF plants. On the other hand, the up-regulation of *GSA, PPO1*, and *CHLD* under inhibition of carotenogenic flux may be derived from the necessity to recover impaired chloroplast biogenesis during photooxidative stress. Our study demonstrates that perturbations in carotenoid and porphyrin biosynthesis coordinate the expression of their biosynthetic genes to sustain plastid function at optimal levels by regulating their metabolic flux in plants under adverse stress conditions.

## Introduction

Chlorophylls and carotenoids are the most widely distributed pigments and essential for ubiquitous structural components of the photosynthetic apparatus. Tetrapyrrole compounds are involved in various metabolic processes such as energy transfer, signal transduction, and catalysis (Dailey, 1990; Tanaka and Tanaka, 2007; Czarnecki et al., 2011). Carotenoid pigments assist in harvesting light energy, function as photo-protectants and antioxidants, and protect the photosynthetic apparatus by quenching harmful reactive oxygen species (ROS) produced by overexcitation of chlorophylls (Krinsky, 1989; Havaux, 1998; Joyard et al., 2009; Murchie and Niyogi, 2011). Their metabolic pathways of synthesis and degradation are tightly controlled to provide adequate amounts of each metabolite (carotenoids/tetrapyrroles) (Cornah et al., 2003; Tanaka and Tanaka, 2007; Lätari et al., 2015; Chayut et al., 2017) and avoid the phototoxic accumulation of intermediates (tetrapyrroles) (Phung et al., 2011).

**FIGURE 1:**
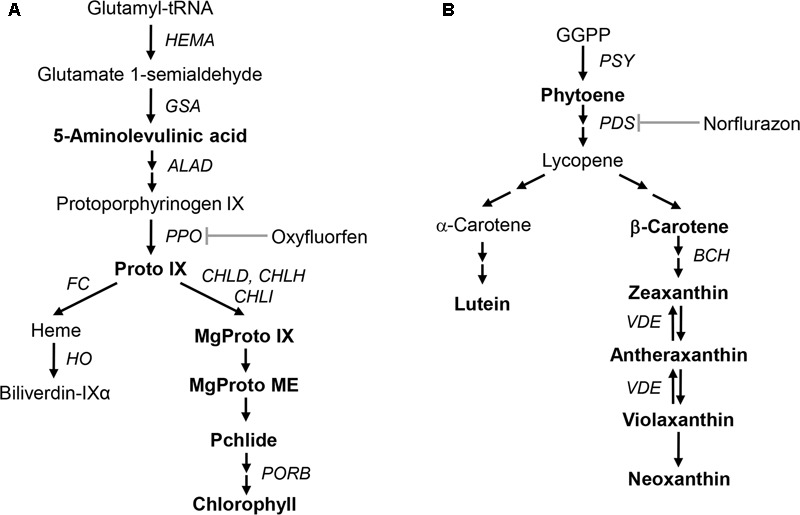
Schematic biosynthetic pathways of porphyrin and carotenoid in plants showing intermediates and genes analyzed in this study. **(A)** Porphyrin biosynthetic pathway. **(B)** Carotenoid biosynthetic pathway. Intermediates quantified in this study are marked in bold. Intermediates: Proto IX, protoporphyrin IX; MgProto IX, Mg-protoporphyrin IX; MgProto ME, Mg-protoporphyrin IX methyl ester; Pchlide, protochlorophyllide; GGPP, geranylgeranyl diphosphate. Genes and enzymes that correspond to the gene names: *HEMA*, glutamyl-tRNA reductase; *GSA*, glutamate 1-semialdehyde aminotransferase; *ALAD*, ALA dehydratase; *PPO*, protoporphyrinogen oxidase; *FC*, Fe-chelatase; *HO*, heme oxygenase; *CHLD*, D-subunit of Mg-chelatase; *CHLH*, H-subunit of Mg-chelatase; *CHLI*, I-subunit of Mg-chelatase; *PORB*, protochlorophyllide oxidoreductase; *PSY*, phytoene synthase; *PDS*, phytoene desaturase; *BCH*, β-carotene hydroxylase; *VDE*, violaxanthin de-epoxidase.

The metabolic pathway from 5-aminolevulinate (ALA), the first committed step, to tetrapyrroles is required for the formation of chlorophyll and heme (**Figure 1**; Beale and Weinstein, 1990; Tanaka and Tanaka, 2007). Protoporphyrinogen IX oxidase (PPO) oxidizes protoporphyrinogen IX (Protogen IX) to form protoporphyrin IX (Proto IX). The first step of the chlorophyll branch involves insertion of the Mg^2+^ into Proto IX by Mg-chelatase, while Fe-chelatase (FC) inserts Fe^2+^ into Proto IX to form protoheme (Terry et al., 2002). Diphenyl ether herbicide oxyfluorfen (OF) is an inhibitor of PPO enzyme in the chloroplast membrane. Accumulated Protogen IX leaks from the plastid and is converted to Proto IX, a photosensitizing compound that promotes photodynamic stress and non-enzymatic lipid peroxidation, by non-specific peroxidases in the cytoplasm (Duke et al., 1991; Jacobs and Jacobs, 1993).

The first committed step in carotenoid biosynthesis is the condensation of two geranylgeranyl diphosphate (GGPP) moieties, yielding the colorless carotene phytoene in a reaction catalyzed by phytoene synthase (PSY) (**Figure 1**; Li et al., 2008; Nisar et al., 2015; Zhou et al., 2015). Phytoene desaturase (PDS) and ζ-carotene desaturase introduce four double bonds into phytoene to form lycopene (Isaacson et al., 2002; Yu et al., 2011). Subsequent cyclization and hydroxylation reactions take place to produce α-carotene and β-carotene. Zeaxanthin, an important component of the xanthophyll cycle, is synthesized by either conversion from β-carotene or de-epoxidation from violaxanthin and is related to the heat dissipation of excess light energy under high-light stress (Brestic et al., 1995; Adams et al., 2006; Lichtenthaler, 2007; Johnson et al., 2008). Bleaching herbicide norflurazon (NF) blocks phytoene desaturation, accumulating phytoene at the expense of colored cyclic carotenoids and leading to typical bleaching symptoms (Böger and Sandmann, 1998).

Distinct photooxidation patterns by NF and OF, which are biosynthetic inhibitors of the carotenoid and porphyrin biosynthetic pathways, coordinate the expression of nuclear- and plastid-encoded photosynthetic genes, thereby differentially altering the structure and function of the photosynthetic apparatus (Park and Jung, 2017). It has been shown that impaired carotenogenesis in carotenoid-deficient mutant and NF-treated plants affects the regulation of carotenoid or porphyrin biosynthesis (Moulin et al., 2008; Aluru et al., 2009; Czarnecki et al., 2011). However, the effect of perturbed porphyrin biosynthesis on the regulation of genes involved in the carotenoid biosynthetic pathway has not been characterized.

In the present study, we examined how distinct photooxidations caused by NF and OF coordinately regulate carotenoid and porphyrin biosynthesis, possibly leading to communication between the two biosynthetic pathways. To address these questions, we analyzed the profiles of metabolites and expression of their biosynthetic genes in the carotenoid and porphyrin pathways under differential photooxidation conditions. Our study demonstrates the importance of tight coordination between the carotenoid and porphyrin biosynthetic pathways, which plays an important role in photoprotection of plants against photodamage and in chloroplast biogenesis.

## Materials and Methods

### Plant Material and Herbicide Treatments

Germinated seeds of rice (*Oryza sativa* cv. Dongjin) plants were sown in pots and grown for 3 weeks in a greenhouse at 25–30°C. Three-week-old plants were transferred to a growth chamber maintained at day/night temperatures of 30/25°C under a 14-h-light/10-h-dark cycle with 500 μmol m^-2^ s^-1^ photosynthetically active radiation. Technical-grade NF (Sigma–Aldrich, St. Louis, MO, United States) and OF (KyungNong, Inc., Seoul, South Korea) were dissolved in 30% acetone containing 0.1% Tween 20. After 3 days of acclimation in the growth chamber, plants were treated with foliar application of either 50 μM OF or 50 μM NF, placed in the dark for 12 h to allow absorption, and then illuminated. Leaf samples for NF1 and OF1 plants were taken 16 h after NF and OF treatments as described by Park and Jung (2017). For NF2 and OF2 plants, leaf samples were taken 88 and 40 h after NF and OF treatment, respectively. Newly developed leaves in NF plants were taken as 88-h samples, as the typical bleaching by NF was not due to photo-dependent destruction of developed leaves, but rather inhibition of pigment synthesis in newly developing leaves.

### Determination of Porphyrins

To measure porphyrin content, plant tissue was ground in methanol:acetone:0.1 N NaOH (9:10:1, [v/v]) and the homogenate was centrifuged at 10,000 × *g* for 10 min (Lermontova and Grimm, 2006). Porphyrin was separated by high-performance liquid chromatography (HPLC) using a Novapak C_18_ column (4-μm particle size, 4.6 mm × 250 mm, Waters, Milford, MA, United States) at a flow rate of 1 mL min^-1^. Porphyrins were eluted with a solvent system of 0.1 M ammonium phosphate (pH 5.8) and methanol. The column eluate was monitored using a fluorescence detector (2474, Waters) at excitation and emission wavelengths of 400 and 630 nm for Proto IX, 440 and 630 nm for protochlorophyllide (Pchlide), and 415 and 595 nm for MgProto IX and MgProto IX methyl ester (MgProto ME), respectively. Fresh leaf tissue was extracted with 100% acetone and chlorophyll content was spectrophotometrically determined as described by Lichtenthaler (1987).

### ALA-Synthesizing Capacity

ALA-synthesizing capacity was measured as described by Papenbrock et al. (1999). Leaf disks were incubated in 20 mM phosphate buffer containing 40 mM levulinic acid in the light for 6 h. Samples were homogenized, resuspended in 1 mL of 20 mM K_2_HPO_4_/KH_2_PO_4_ (pH 6.9), and centrifuged at 10,000 × *g*. The 500-μL supernatant was mixed with 100 μL ethyl acetoacetate, boiled for 10 min, and cooled for 5 min. An equal volume of modified Ehrlichs reagent was added and the absorption of the chromophore was determined at 553 nm with a spectrophotometer.

### Enzyme Activities of Porphyrin Biosynthesis

To evaluate PPO, leaves or roots were homogenized in homogenization buffer [0.5 M sorbitol, 0.1 M Tris-HCl, pH 7.5, 1 mM DTT, and 0.1% bovine serum albumin (BSA)]. The mixture was centrifuged at 5,000 × *g* for 10 min. The crude chloroplast pellet was resuspended in assay buffer (0.1 M Tris-HCl, pH 7.5, 5 mM DTT, 1 mM EDTA, and 0.03% Tween 80). The substrate Protogen IX was prepared by chemical reduction of Proto IX with sodium mercury amalgam (Sigma–Aldrich). PPO activity was determined as described by Lermontova and Grimm (2000). The enzyme reaction was incubated at 30°C for 5 min and stopped by adding ice-cold methanol:DMSO (8:2, [v/v]). Proto IX was separated by HPLC as described above.

Mg-chelatase was assayed as described by Lee et al. (1992), with some modifications. Leaf tissue was homogenized in homogenization buffer consisting of 0.5 M sorbitol, 50 mM Tricine, pH 7.8, 0.1% BSA, 1 mM MgCl_2_, and 1 mM DTT, and then centrifuged at 5,000 × *g* for 10 min. Crude plastids were incubated in homogenization buffer (without BSA) containing 4 mM MgATP in a regenerating system (60 mM phosphocreatine/creatine phosphokinase, 10 units mL^-1^) and 10 mM MgCl_2_. Reactions were started by adding DMSO-solved Proto IX to a final concentration of 100 μM, incubated at 30°C for 60 min, and stopped by adding cold acetone. The MgProto IX in hexane-washed water-acetone extracts was evaluated by fluorescence detection at excitation and emission wavelengths of 415 and 595 nm.

Fe-chelatase activity was measured using the protocol from Papenbrock et al. (1999). Crude plastids from leaves or roots were lysed in 0.1 M Tris-HCl buffer (pH 7.3), 0.5% Tritone X-100, and 1 mM DTT, and membranes were centrifuged, resuspended in the same buffer, and re-centrifuged. Two hundred-microliter aliquots of the supernatant were mixed with 4 μL of 6 mM DMSO-solved Proto IX, 2 μL of 0.5 M ZnSO_4_, and 2 μL of 100 mM palmitic acid in the dark at 30°C for 45 min. Assay samples were extracted twice with acetone: 0.1 N NH_4_OH (9:1, [v/v]). The ZnProto in hexane-washed water-acetone extracts was evaluated by fluorescence detection at excitation and emission wavelengths of 416 and 589 nm.

### Determination of Carotenoids

For carotenoid analysis, leaf tissues were ground in 100% acetone containing 10 mg CaCO_3_. The extracts were centrifuged at 16,000 × *g* for 10 min and the resulting supernatants were collected. The pigments were separated by HPLC as previously described by Gilmore and Yamamoto (1991) using a Waters 2690 System (Waters) equipped with a Waters 2487 Absorbance Detector (Waters). A Spherisorb ODS-1 column (5-μm particle size, 250 mm × 4.6 mm id) was obtained from Alltech, Inc. (Deerfield, IL, United States). Solvent A (acetonitrile:methanol:Tris-HCl buffer 0.1 M pH 8.0, 72:8:3, v/v/v) was run isocratically from 0 to 4 min followed by a 2.5-min linear gradient to 100% solvent B (methanol-hexane, 4:1, v/v) at flow rate of 2 mL min^-1^. HPLC runs were carried out at 445 nm for xanthophylls and β-carotene and at 285 nm for phytoene.

### RNA Extraction and qRT-PCR

Total RNA was prepared from leaf tissues using TRIZOL Reagent (Invitrogen, Carlsbad, CA, United States), and 5 μg of RNA from each sample was used for the reverse transcription reaction (SuperScript III First-Strand Synthesis System, Invitrogen). Subsequently, 50 ng of cDNA was used for qRT-PCR analysis. The qRT-PCR analysis was carried out in the StepOnePlus Real-Time PCR system (Applied Biosystems, Foster City, CA, United States) using Power SYBR Green PCR Master Mix (Applied Biosystems) and primers specific for each (Supplementary Table S1). The qRT-PCR program consisted of 2 min at 50°C, 10 min at 95°C, and 40 cycles of 15 s at 95°C and 1 min at 60°C. Melting curve analysis was performed after every PCR to confirm the accuracy of each amplified product. All reactions were evaluated in triplicate. A control sample was used for calibration, with the expression level of the sample set to 1. Actin was used as an internal control.

### Statistical Analysis

Data were expressed as the means ± SE. Differences were analyzed by the Duncan’s multiple range test. *P*-values < 0.05 were considered significant. The analyses were performed using SPSS software (SPSS, Inc., Chicago, IL, United States).

## Results

### Effects of Differential Photooxidative Stress on Porphyrin Intermediates in Rice Plants Treated with NF and OF

**FIGURE 2:**
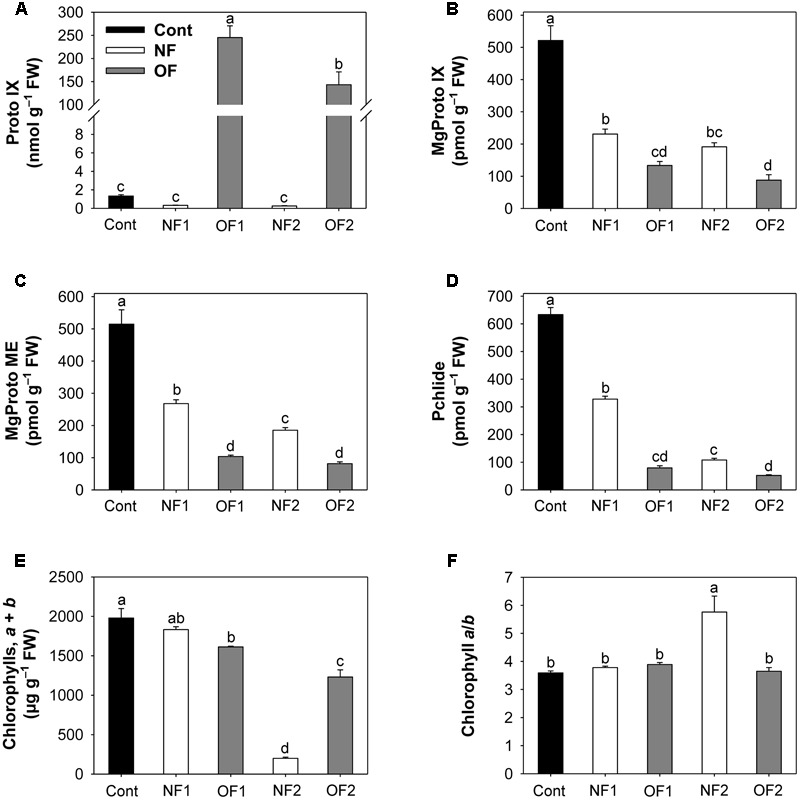
Photooxidation-induced changes in levels of porphyrin intermediates in rice plants treated with either NF or OF. **(A)** Proto IX. **(B)** MgProto IX. **(C)** MgProto ME. **(D)** Pchlide. **(E)** Chlorophylls, *a* + *b*. **(F)** Chlorophyll *a*/*b*. Three-week-old rice plants were treated with either 50 μM OF or 50 μM NF. Cont, control; NF1 and NF2, 16 and 88 h after 50 μM NF treatment, respectively; OF1 and OF2, 16 and 40 h after 50 μM OF treatment, respectively. The data represent the mean ± SE of nine replicates from three independent experiments. Means denoted by the same letter did not differ significantly at *P* < 0.05 according to Duncan’s multiple range test.

To investigate the effect of differential photooxidative stress on porphyrin biosynthesis, we examined photooxidation-induced changes in levels of porphyrin biosynthetic intermediates. High levels of Proto IX accumulated in response to OF treatment, with a greater increase in OF1, whereas Proto IX decreased in NF-treated plants (**Figure 2A** and Supplementary Figure S1). Both NF and OF treatments resulted in greater decreases in the intermediates of the Mg-porphyrin branch including MgProto IX, MgProto ME, and Pchlide, with greater decreases observed in OF-treated plants (**Figures 2B–D**). NF2 plants showed the greatest decrease in the content of chlorophyll, which is the end product of the Mg-porphyrin branch, whereas OF2 plants showed only a slight decrease (**Figure 2E**). The chlorophyll *a*/*b* ratio increased markedly in NF2 plants, but not in OF2 plants (**Figure 2F**). Our results showed that NF, which impairs the carotenogenesis and photosynthetic apparatus, and the deregulator of porphyrin biosynthesis, OF, resulted in large perturbations in porphyrin biosynthesis.

### Differential Photooxidations Dramatically Affect Gene Expression and Enzyme Activity in the Porphyrin Metabolic Pathway

**FIGURE 3:**
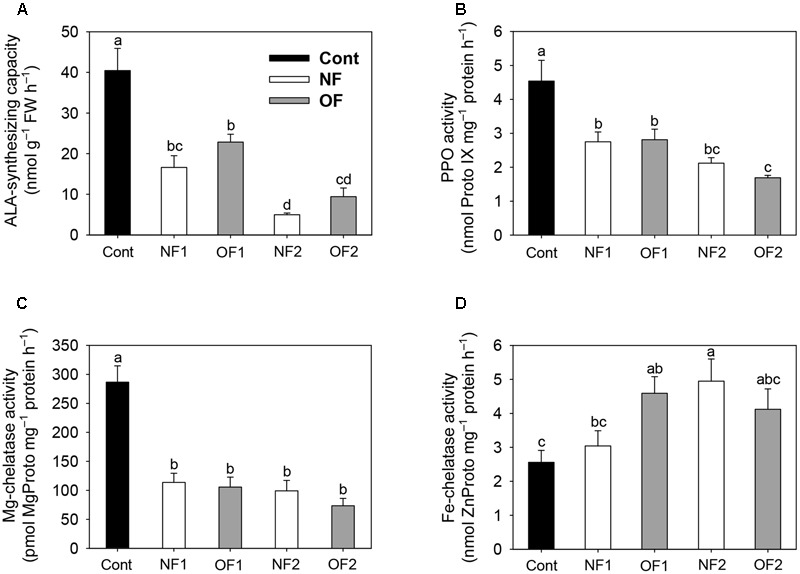
Effect of differential photooxidation on porphyrin-synthesizing enzyme activity in rice plants treated with either NF or OF. **(A)** ALA-synthesizing capacity. Leaf disks were incubated in 20 mM phosphate buffer containing 40 mM levulinic acid in the light for 6 h. **(B)** PPO activity. **(C)** Mg-chelatase activity. **(D)** Fe-chelatase activity. The plants were subjected to the same treatments as in **Figure 2**. Treatment notations are the same as in **Figure 2**. The data represent the mean ± SE of nine replicates from three independent experiments. Means denoted by the same letter did not differ significantly at *P* < 0.05 according to Duncan’s multiple range test.

**FIGURE 4:**
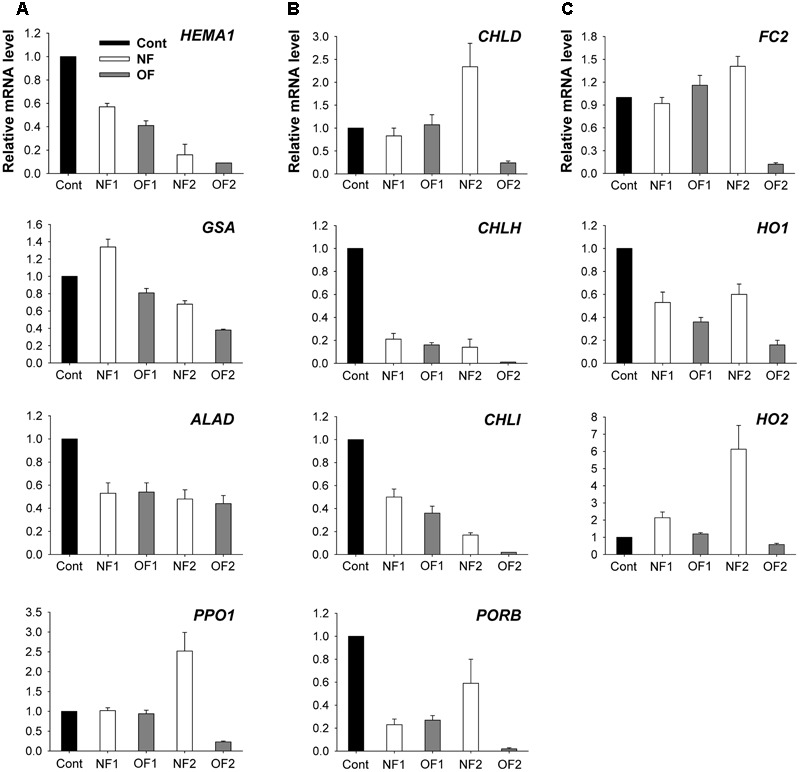
Photooxidation-induced changes in expression of genes encoding enzymes of the porphyrin biosynthetic pathway. **(A)** Common branch. **(B)** Mg-porphyrin branch. **(C)** Fe-porphyrin branch. The plants were subjected to the same treatments as in **Figure 2**. Treatment notations are the same as in **Figure 2**. Total RNAs were purified from plants and reverse-transcribed. The resulting cDNAs were used as templates for qRT-PCR using the actin gene as an internal control. The untreated control was used for normalization, with the expression level of the sample set to 1. The data represent the mean ± SE of nine replicates from three independent experiments.

To examine the molecular mechanisms underlying the photooxidation-induced changes in porphyrin levels, we analyzed the expression of enzymes involved in porphyrin biosynthesis by using enzyme assays and qRT-PCR. The capacity of plants to synthesize ALA, a key precursor in the pathway, was compared under differential photooxidative conditions caused by NF and OF. The ALA-synthesizing capacity decreased greatly 1 day after NF and OF treatments compared to that of the control, and then decreased further in later stages of treatments (**Figure 3A**). This data shows that the plants could not maintain the capacity to accumulate ALA under progressive photooxidative stress, with a greater decrease observed in NF plants. We also assayed the photooxidation-dependent expression of genes involving ALA-synthesizing activity in the early biosynthetic steps before the branch point. The transcript levels of *HEMA1* which encodes glutamyl-tRNA reductase began decreasing at 16 h after NF and OF treatments, and decreased further in NF2 and OF2 plants (**Figure 4A**). Although the transcript levels of glutamate 1-semialdehyde aminotransferase (*GSA*) increased in NF1 plants and then decreased slightly in NF2 plants, they gradually decreased with progressive inhibition with OF. *ALAD*, which encodes the enzyme that converts ALA to porphobilinogen, was down-regulated with both NF and OF treatments. In response to OF treatment, the activities of PPO, the enzyme that catalyzes the last step before the branch point, and target site of OF inhibition, decreased gradually in treated plants *(***Figure 3B**). Transcript levels of *PPO1* also greatly decreased with progressive inhibition of PPO activity in OF2 plants. Interestingly, NF2 plants exhibited a 2.5-fold increase in the transcript levels of *PPO1*, although they showed significantly decreased PPO activity (**Figure 4A**).

As photooxidative stress markedly affected the levels of Mg-porphyrin intermediates, we examined the activities of two enzymes at the branch point, Mg- and Fe-chelatase. In addition to their lower PPO activities, both NF and OF plants maintained lower activities of Mg-chelatase, which inserts Mg^2+^ into Proto IX in the Mg-porphyrin branch, compared to control plants (**Figure 3C**). Among the genes encoding the three Mg-chelatase subunits *CHLH, CHLI*, and *CHLD*, transcript levels of *CHLD* greatly increased in NF2 plants, but decreased in OF2 plants (**Figure 4B**). Transcript levels of *CHLH* and *CHLI* greatly decreased in NF and OF plants, with the greatest decrease observed in OF2 plants. Expression of *PORB* encoding the enzyme that generates chlorophyllide from Pchlide was greatly down-regulated in NF1 and OF1 plants, whereas it recovered to some extent in NF2 plants, but further decreased in OF2 plants.

In the Fe-porphyrin branch, the enzyme activities of FC were noticeably increased in NF and OF plants during the progression of photooxidative stress (**Figure 3D**). Transcript levels of *FC2* also increased by 16 and 41% in OF1 and NF2 plants, respectively, compared to their controls, but decreased greatly in OF2 plants (**Figure 4C**). Transcript levels of *HO1* encoding heme oxygenase (HO), which catalyzes the formation of biliverdin-IXα from heme, decreased in NF and OF plants, with greater decreases in OF plants. In response to NF treatment, the transcript levels of *HO2* markedly increased, showing a sixfold increase in NF2 plants. In contrast, the transcript levels of *HO2* slightly increased in OF1 plants, but decreased in OF2 plants.

### Effects of Differential Photooxidative Stress on Intermediates and Gene Expression in the Carotenoid Biosynthetic Pathway

Phytoene, the precursor of the carotenoid biosynthetic pathway, was not detected in control and OF plants, whereas it drastically accumulated in plants treated with NF, which blocks carotenoid synthesis by specifically inhibiting *PDS* (**Table 1** and Supplementary Figure S2). As a result, the levels of lutein, β-carotene, violaxanthin, and neoxanthin were greatly decreased after 88 h of NF treatment, which may have caused bleaching of the newly emerged parts of the leaves, while their levels remained nearly constant in response to OF treatment compared to the control. In contrast, zeaxanthin and antheraxanthin, which have photoprotective functions, were newly synthesized in response to the inhibition of carotenoid biosynthesis by NF, whereas they were not detected in plants treated with OF (**Table 1**).

**Table 1:** Photooxidation-induced changes in levels of carotenoid biosynthetic intermediates in rice plants treated with either NF or OF.

	Phytoene^1^	Lutein	β-Carotene	Zeaxanthin nmol g^-1^ FW	Antheraxanthin	Violaxanthin	Neoxanthin
**Treatments**							
Cont	N.D.	386 ± 30a	548 ± 42a	N.D.	N.D.	333 ± 23a	225 ± 17ab
NF1	575 ± 84b	359 ± 12a	410 ± 27b	80 ± 3b	55 ± 5a	182 ± 5b	189 ± 2b
OF1	N.D.	390 ± 21a	562 ± 19a	N.D.	N.D.	351 ± 13a	237 ± 10a
NF2	2091 ± 121a	143 ± 6b	62 ± 5c	89 ± 4a	24 ± 3b	24 ± 3c	70 ± 4c
OF2	N.D.	366 ± 23a	545 ± 46a	N.D.	N.D.	310 ± 15a	222 ± 16ab

*The plants were subjected to the same treatments as in **Figure 2**. Treatment notations are the same as in **Figure 2**. N.D., not detectable. ^1^Relative level of phytoene is expressed as a peak area. The data represent the mean ± SE of six replicates from two independent experiments. Within each column, means denoted by the same letter did not differ significantly at *P* < 0.05 according to Duncan’s multiple range test.*

**FIGURE 5:**
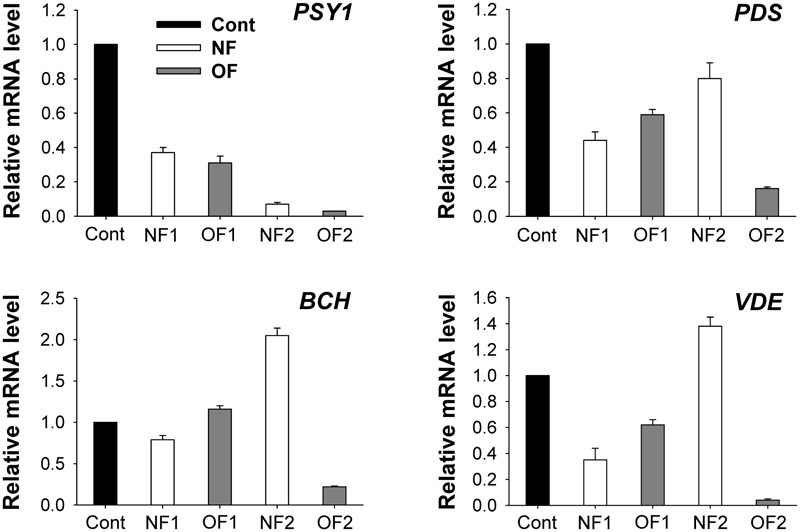
Photooxidation-induced changes in expression of genes encoding enzymes of the carotenoid biosynthetic pathway. The plants were subjected to the same treatments as in **Figure 2**. Treatment notations are the same as in **Figure 2**. Total RNAs were purified from plants and reverse-transcribed. The resulting cDNAs were used as templates for qRT-PCR using the actin gene as an internal control. The untreated control was used for normalization, with the expression level of the sample set to 1. The data represent the mean ± SE of nine replicates from three independent experiments.

Phytoene synthase and phytoene desaturase are the first two regulatory enzymes in the carotenoid biosynthetic pathway. Transcript levels of *PSY1* greatly decreased in response to NF and OF treatments, with greater decreases detected in NF2 and OF2 plants (**Figure 5**). Plants showed greatly decreased transcript levels of *PDS* after 16 h of 50 μM NF, which then increased to a level similar to those of the control after 88 h of treatment. Transcript levels of *BCH* encoding β-carotene hydroxylase (BCH), which catalyzes the conversion of β-carotene to zeaxanthin, increased by twofold in NF2 plants, whereas they slightly increased in OF1 plants and then greatly decreased in OF2 plants. Violaxanthin de-epoxidase (VDE), a key step in the xanthophyll cycle, catalyzes the conversion from violaxanthin to zeaxanthin through antheraxanthin to protect the photosynthetic apparatus. Transcript levels of *VDE* greatly decreased in NF1 plants, but increased by 38% in NF2 plants compared to those of control plants. Transcript levels of *PDS* and *VDE* gradually decreased with the progression of photooxidative stress caused by OF treatment (**Figure 5**).

## Discussion

Norflurazon caused severe disruption in thylakoid membranes and bleaching symptom on the leaves of treated plants, whereas plants treated with OF exhibited disruption of the chloroplast envelope and plasma membrane, rapidly leading to an apparent necrotic phenotype (Park and Jung, 2017). In addition, NF plants undergo more severe photooxidative damage compared to OF plants, as indicated by the greater decline in the photosynthetic efficiency (*F*_v_/*F*_m_), disruption of lamella bodies, and reduction of light-harvesting chlorophyll-binding proteins (Park and Jung, 2017). The distinct photooxidation patterns and chloroplast status imposed by inhibitors of carotenoid and porphyrin biosynthesis may differentially regulate the expression of their biosynthetic genes under stress conditions, possibly enabling the plant to coordinate the two pathways.

Many intermediates in the porphyrin biosynthetic pathway interact with light, generating ROS such as ^1^O_2_, which is harmful to the cell and causes peroxidation of membrane lipids (Kouji et al., 1988; Lehnen et al., 1990). The photosensitization reaction of greatly accumulated Proto IX in response to OF treatment (**Figure 2A**) may contribute to the necrotic spots observed in treated plants by affecting the membranes adjacent to the cytosol, i.e., chloroplast envelope and plasma membrane (Park and Jung, 2017). The bleaching symptoms and decreased levels of Proto IX in NF2 plants (**Figure 2A**) appeared to result from the arrest of plastid development due to the failure of thylakoid membrane assembly. Both NF and OF treatments markedly decreased the levels of MgProto IX, MgProto ME, and Pchlide, with greater decreases observed in OF-treated plants (**Figure 2B**). Upon illumination, porphyrins such as Proto IX and Pchlide may be involved in the modulated response to oxidative stress or programmed cell death (Kariola et al., 2005; van Lis et al., 2005; Yao and Greenberg, 2006; Kilinc et al., 2009). NF2 plants showed not only great decreases in chlorophyll, the end product of the porphyrin pathway, but also marked increases in the chlorophyll *a*/*b* ratio (**Figures 2E,F**), which is considered an adaptive feature to reduce the possibility of photooxidative damage to the photosynthetic apparatus (Jung, 2004). Unlike OF, which directly inhibits porphyrin biosynthesis, NF does not inhibit porphyrin synthesis. However, the absence of carotenoids may impair plastid components, mainly by enhancing the photosensitizing properties of porphyrin intermediates.

Tight control of porphyrin biosynthesis ensures sufficient enzymatic capacity for each step in their synthesis to avoid excessive accumulation of porphyrins (Lermontova and Grimm, 2006). Both NF- and OF-treated plants could not maintain the capacity to synthesize ALA, an early precursor in porphyrin biosynthesis, under progressive photooxidative stress (**Figure 3A**). This indicates that perturbed metabolic flow in either porphyrin or carotenoid biosynthesis significantly alters the control of ALA biosynthesis. Photooxidation caused by NF and OF also induced changes in the expression of a large set of genes involved in porphyrin biosynthesis. Expression of genes involving ALA-synthesizing activity including *HEMA, GSA*, and *ALAD* was greatly down-regulated in plants treated with OF (**Figure 4A**). However, not all genes involved in ALA synthesis were strongly down-regulated after NF treatment; for example, the transcript levels of *GSA* were increased in NF1 plants. Strong repression of *HEMA1* was also observed in *Arabidopsi*s seedlings treated with NF (Moulin et al., 2008). Interestingly, expression of *PPO1* dramatically increased in NF2 plants, although the activities of PPO, the enzyme that catalyzes the last step before the branch point, was decreased significantly in NF plants (**Figures 3B, 4A**). Additionally, the transcript levels of *PPO1* and activity of PPO decreased with photooxidation progression in OF2 plants. The up-regulation of *GSA* and *PPO1* in NF plants may be an effort to compensate for their downstream components in the porphyrin biosynthetic pathway under photooxidative stress.

In addition to the lower PPO activities, exposure to either NF or OF which causes photooxidative stress resulted in strong inactivation of Mg-chelatase, a key enzyme in the Mg-porphyrin branch, compared to control plants (**Figure 3C**). The expression levels of *CHLH* and *CHLI* encoding Mg-chelatase subunits were significantly down-regulated in NF and OF plants, while the expression levels of *CHLD* exhibited a 2.3-fold increase in NF2 plants and decrease in OF2 plants (**Figure 4B**). Transcript levels of *PORB*, which is crucial for the photoconversion of protochlorophyllide to chlorophyllide (Masuda et al., 2003), greatly decreased in NF1 plants and then recovered to some extent in NF2 plants, whereas they gradually decreased in OF plants. These data are consistent with the observation that both NF and OF plants had a markedly reduced capacity to synthesize chlorophylls and Mg-porphyrin intermediates (**Figure 2**). Under photooxidative stress conditions, rapid shut-down of porphyrin biosynthesis may be necessary to reduce the synthesis of potentially dangerous Mg-porphyrins. Strong repression of genes in the Mg-porphyrin branch under plant stress conditions has also been observed in other studies (Moulin et al., 2008; Phung et al., 2011).

During the progression of photooxidative stress, increased activities of FC in the Fe-porphyrin branch were detected in NF and OF plants (**Figure 3D**), further supporting that FC plays an important role in the plant cell’s ability to overcome oxidative stress. Both NF2 and OF1 plants showed increased transcript levels of *FC2* by 41 and 16%, respectively (**Figure 4C**), compared to control plants. This up-regulation of *FC2* may contribute to the production of hemoproteins that are required in response to severe oxidative stress. The discrepancy between the enzyme activity and transcript levels of FC in OF2 plants indicates the post-translational regulation of the enzyme. In white *albostrians* leaves of barley mutant, which is similar to bleaching in NF plants, FC activity was threefold higher than in green leaves and correlated with enhanced accumulation of FC (Yaronskaya et al., 2003). Plant HOs also play a role in protection against oxidative cell damage (Noriega et al., 2004; Bellner et al., 2009; Kim et al., 2014). Unlike the decreased expression of *HO1* in response to NF and OF, the expression levels of *HO2* were greatly induced during photooxidative stress, showing a sixfold increase in NF2 plants (**Figure 4C**). Our results demonstrate that porphyrin synthesis and degradation are carefully adjusted to reflect different cellular needs under distinct photooxidative stress imposed by NF and OF, which has been previously observed under varying environmental conditions (Reinbothe and Reinbothe, 1996; Phung et al., 2011; Kim et al., 2014; Phung and Jung, 2015).

Carotenoids are essential for chloroplast biogenesis by preventing photoinhibition during photosynthesis (Wu et al., 1999; Carol and Kuntz, 2001). PSY, which catalyzes the first committed condensation step from GGPP to produce phytoene, is the key regulatory enzyme in carotenogenesis in plastids (Li et al., 2008; Nisar et al., 2015; Zhou et al., 2015). The gradual decrease in transcript levels of *PSY1* in response to NF and OF treatments (**Figure 5**) was similar to the repression of *PSY* in *Arabidopsis immutans* white leaf sectors. NF blocks PDS, leading to the accumulation of colorless phytoene at the expense of colored cyclic carotenoids (Böger and Sandmann, 1998). In control and OF-treated plants, the precursor phytoene was below the detection limit, whereas it excessively accumulated in NF-treated plants (**Table 1**), indicating that PDS activity was inhibited. Transcript levels of *PDS* decreased in both NF- and OF-treated plants, although its transcript level in NF2 recovered to a level similar to that of the control (**Figure 5**), which may be necessary to compensate for the inhibited PDS activity.

Without colored carotenoids, plastids experience severe photooxidative stress, even under lower light (Oelmüller and Mohr, 1986; Voigt et al., 2010). NF treatment greatly decreased the contents of colored carotenoids including lutein, neoxanthin, violaxanthin, and β-carotene, whereas their levels remained nearly constant in response to OF treatment (**Table 1**). In addition to their functions in photoprotection, carotenoids play a role in the sensing and signaling of photooxidative stress (Ramel et al., 2012; Zhang et al., 2013). The significant decrease in β-carotene and violaxanthin in NF2 plants may partly contribute to new formations of antheraxanthin and zeaxanthin in NF2 plants under photooxidative conditions (**Table 1**). The transcript levels of *BCH* encoding BCH, which catalyzes the conversion of β-carotene to zeaxanthin, greatly increased in NF2 plants, but greatly decreased in OF2 plants (**Table 1** and **Figure 5**). Increased expression of the BCH enzyme increases the content of zeaxanthin in the chloroplast membrane (Davison et al., 2002). Transcript levels of *VDE*, which encodes a key enzyme in the xanthophyll cycle, in NF2 and OF2 plants exhibited similar expression patterns as in those of *BCH* (**Figure 5**). Photosynthetic apparatus responds not only with a quick de-epoxidation of violaxanthin to zeaxanthin, but also with a fast *de novo* biosynthesis of zeaxanthin under high irradiance stress (Lichtenthaler, 2007). The de-epoxidation reaction by VDE contributes to non-photochemical quenching of energy to dissipate excess light energy (Brestic et al., 1995; Niyogi, 1999; Jahns and Holzwarth, 2012; Ballottari et al., 2014). Up-regulation of *BCH* and *VDE* as well as increased levels of antheraxanthin and zeaxanthin triggered by NF are likely critical against preceding photooxidative stress in chloroplasts.

Carotenoids are commonly known to function in chloroplast biogenesis, antioxidants, and protection of the photosynthetic apparatus by quenching harmful ROS according to many previous studies (Krinsky, 1989; Havaux, 1998; Lichtenthaler, 2007; Murchie and Niyogi, 2011). However, the protective role of carotenoids against the destructive effects of photosensitizing molecules, i.e., porphyrin intermediates, has not been well-characterized in plants. NF greatly decreased the levels of intermediates and gene expression in the Mg-porphyrin branch, which may be because even low levels of porphyrin can trigger cell damage via photosensitized reactions in the absence of quenching pigments, carotenoids, as well as because of impaired plastid biogenesis. Therefore, plants treated with NF greatly down-regulate most porphyrin genes in the Mg-porphyrin branch to minimize the synthesis of potentially dangerous Mg-porphyrin intermediates. Interestingly, up-regulation of *GSA, PPO1*, and *CHLD* under perturbation of carotenogenesis likely results from the need to repair impaired plastids, indicating that the plastids are active. This view is supported by the normal accumulation of plastid-encoded photosynthetic genes including *RbcL, PsaC*, and *PsbD* in NF-treated plants (Park and Jung, 2017). In contrast, perturbed porphyrin biosynthesis by OF, which keeps thylakoid membranes relatively intact during photooxidative stress (Park and Jung, 2017), did not noticeably influence the levels of carotenoid intermediates, regardless of the strong down-regulation of carotenoid biosynthetic genes. In addition to the sustained levels of carotenoids, the marked scavenging of Mg-porphyrins that occurs from greatly down-regulating their biosynthetic genes in OF-treated plants may not require the additional biosynthesis of carotenoids.

## Conclusion

Perturbations in carotenoid and porphyrin biosynthesis coordinate the expression of their biosynthetic genes, which are essential for chloroplast biogenesis, to maintain chloroplast function at optimum levels by regulating their metabolic flux accordingly. Distinct photooxidation patterns by NF and OF resulted in enhanced protection against cellular damage not only through strong scavenging of phototoxic porphyrins, but also through up-regulation of *FC2, HO2*, and Fe-chelatase, particularly with increased levels of zeaxanthin via up-regulation of *BCH* and *VDE* in NF plants. Sensing of altered metabolite levels in one pathway as well as photooxidative status may be responsible for some of the changes in gene expression profiles in the other biosynthetic pathway under differential photooxidative stress, in which plants develop a distinct photobleaching or necrosis phenotype. This study provides insight into the underlying molecular mechanisms of photosynthetic pigment synthesis under photooxidative stress.

## Author Contributions

SJ and J-HP designed the experiments. J-HP, LT, and SJ performed the experiments and analyzed the data. SJ wrote the manuscript. All authors approved the final manuscript.

## Conflict of Interest Statement

The authors declare that the research was conducted in the absence of any commercial or financial relationships that could be construed as a potential conflict of interest.
