# Supplementary material for: Perturbations in the Photosynthetic Pigment Status Result in Photooxidation-Induced Crosstalk between Carotenoid and Porphyrin Biosynthetic Pathways
Source: Front Plant Sci. 2017 Nov 20;8:1992. doi: 10.3389/fpls.2017.01992 (PMC5701815; doi:10.3389/fpls.2017.01992)
Supplement: Supplementary file 1 [file Table_1.PDF]

Table S1. Primers used for qRT-PCR assays.

Gene	Primer sequence
HEMA1	F: GCTATGGGTGGTGTTCGACT
	R: CGATCTTCTGGAGGCACTTC
GSA	F: CTCCGTGACTTGACGAAACA
	R: GTAGGTTCCAGGCTCCATCA
ALAD	F: GTCCACCGTCTCCTTCTCC
	R: TGTCAAGTCAAGAGGCCTGA
PPO1	F: ACAGTTCCTCATTGGCCATC
	R: CCCATGAAATTTTTGCTGCT
CHLD	F: TGGGACAGCAAAGACAGTGA
	R: AAGGCCAGGTTGAAACACAG
CHLH	F: GTGTGGGTTGCGTTCTTTTT
	R: GGTGACAATGTGGCTCCTCT
CHLI	F: TGTGCTTCTGGATTCTGCTG
	R: GCTGGAGCTTGTCTTGTTCC
PORB	F: GTGAATTGCCAGGTTTTCGT
	R: GCAATTAGCAAAGCTGCACA
FC2	F: TTGGTGCTATGGCAGTTTCA
	R: AGTGGAACAAAGGCAGGATG
HO1	F: AGCGCTAGCAGTAGCAGGAG
	R: GCTCCTTCTCCCCTTCCTT
HO2	F: AGGGACCTAGCAGCCCTAAC
	R: CCCGTATCGTCCATCTTGAG
PSY1	F: GCCCAACAACAAGGAGAAGA
	R: GATGATAGGCCTGCTTGAGG
PDS	F: TGCAAAGATCACCGATTGAA
	R: GAGAGTTGGGCACCCACTAA

BCH	F: CAACCGGAGCTTGTGATTTT
	R: TACAATGTTCAGCCGCAGAG
VDE	F: GAAATGCGTCCCACAAAAGT
	R: TTATAGAGGATCGCGGGTTG
Actin	F: CTTCATAGGAATGGAAGCTGCGGGTA
	R: CGACCACCTTGATCTTCATGCTGCTA

F, forward; R, reverse.
