# Supplementary material for: Perturbations in the Photosynthetic Pigment Status Result in Photooxidation-Induced Crosstalk between Carotenoid and Porphyrin Biosynthetic Pathways
Source: Front Plant Sci. 2017 Nov 20;8:1992. doi: 10.3389/fpls.2017.01992 (PMC5701815; doi:10.3389/fpls.2017.01992)
Supplement: Supplementary file 2 [file Image_1.PDF]

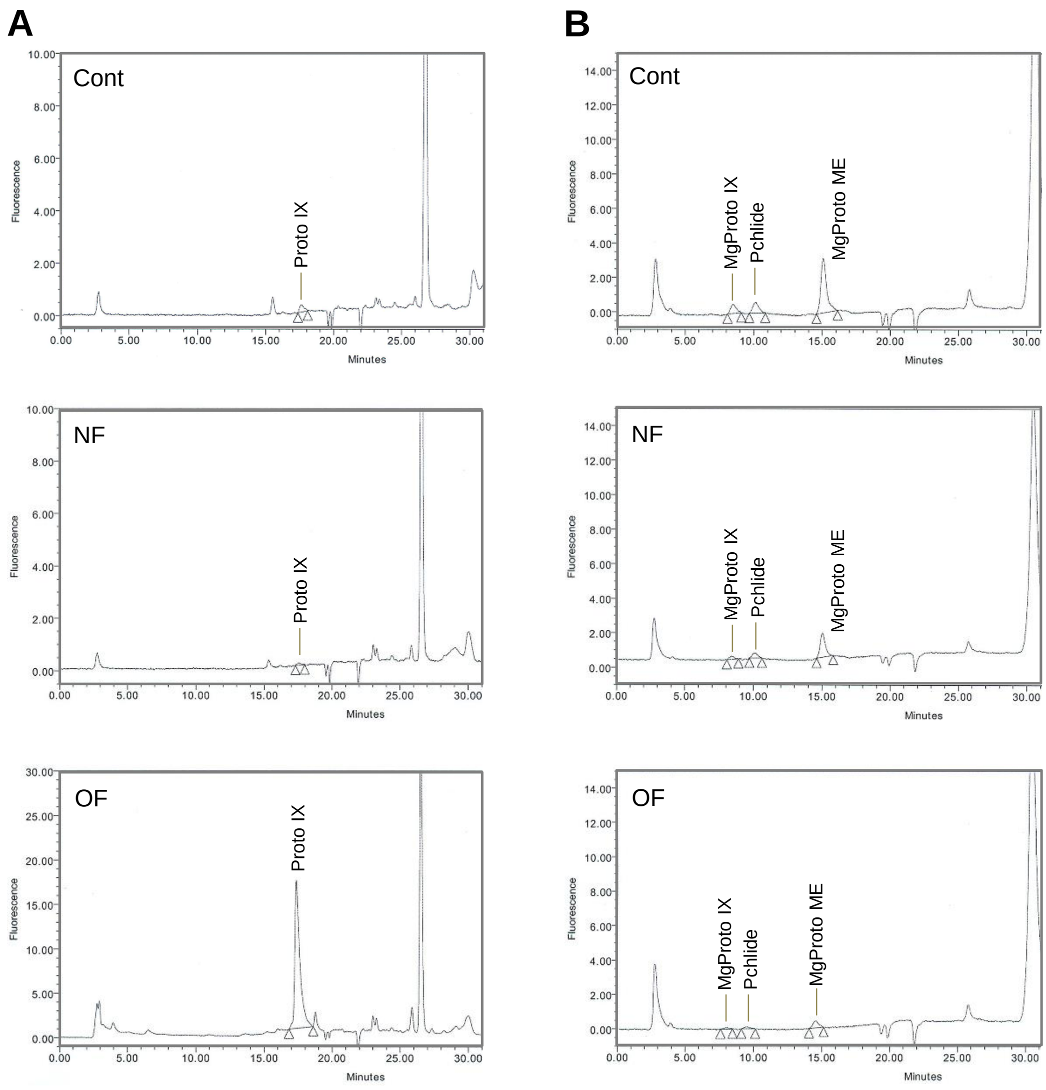

Supplemental Figure S1. HPLC chromatograms of porphyrin intermediates**. A**, Proto IX. **B**, Mg-porphyrins. Cont, control; NF, 88 h after 50 μM NF treatment; OF, 40 h after 50 μM OF treatment.

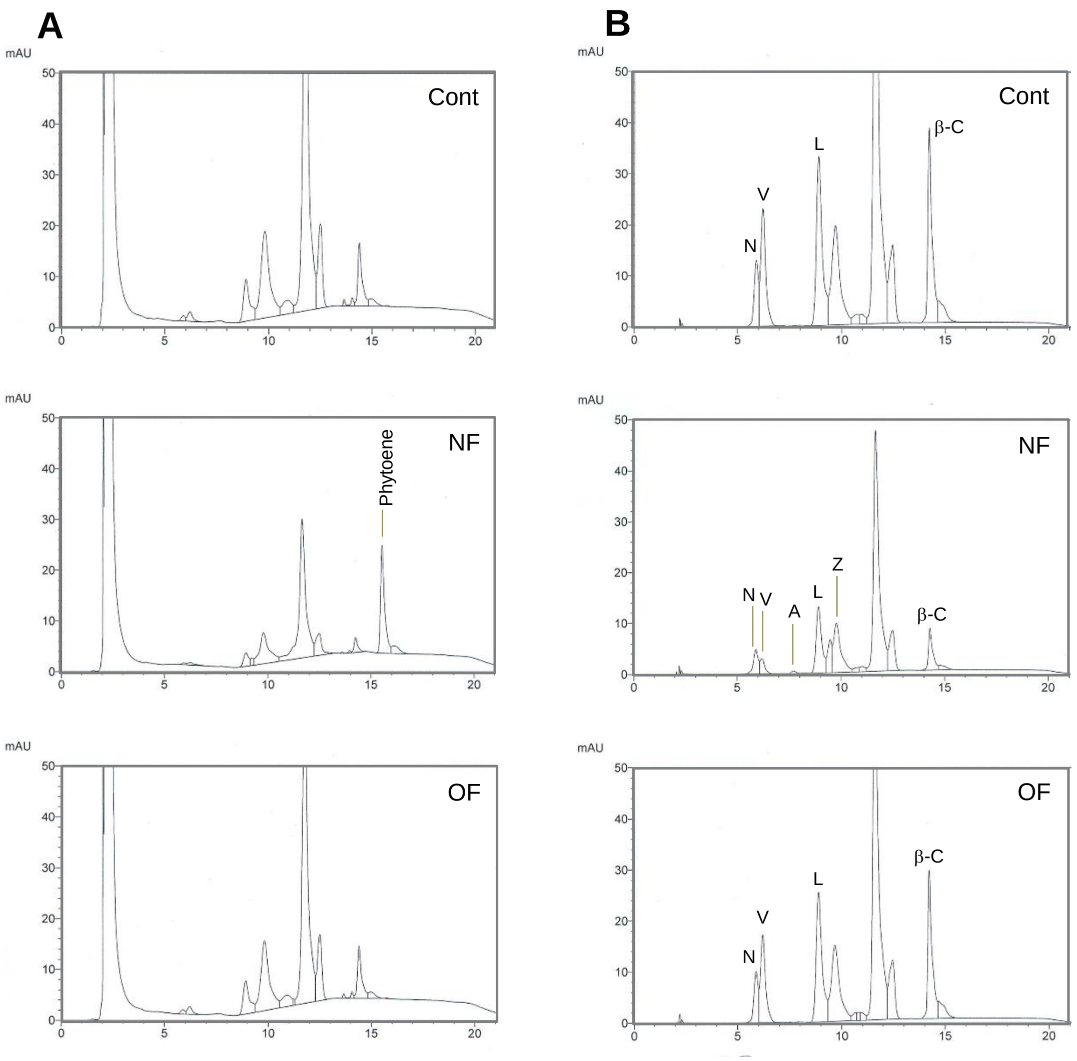

Supplemental Figure S2. HPLC chromatograms of carotenoid intermediates**. A**, Phytoene. **B**, Other carotenoid intermediates. Cont, control; NF, 88 h after 50 μM NF treatment; OF, 40 h after 50 μM OF treatment. N, neoxanthin; V, violaxanthin, A, antheraxanthin; L, lutein; Z, zeaxanthin, β-C, β-Carotene.
